# Supplementary material for: Silencing of a Pectin Acetylesterase (PAE) Gene Highly Expressed in Tobacco Pistils Negatively Affects Pollen Tube Growth
Source: Plants (Basel). 2023 Jan 10;12(2):329. doi: 10.3390/plants12020329 (PMC9864977; doi:10.3390/plants12020329)
Supplement: Supplementary file 1 [file plants-12-00329-s001.zip › Supplementary Figures.pdf]

Supplementary Figures

```
1     ctttacagcttttcaaactcttgttcaacttcttctgctatagaaagagagaaagacaaat  61

62    agcccttaacttattttattgcttctggctgttagaatggagaaagtgatggattgtaac  121
                                          M  E  K  V  M  D  C  N
122   tcaattctcatggctgcagtgctttttctctactgtgtctttggacaacaactgaagga  181
       S  I  L  M  A  A  V  L  F  S  L  L  C  L  W  T  T  T  E  G
182   gacctcattgttaatataacctacctcgagactgccatatctaagggtgcagtatgcttg  241
       D  L  I  V  N  I  T  Y  L  E  T  A  I  S  K  G  A  V  C  L
242   gatggaagtgctccagctttccactttgatcctggaactggttcaggaatttatagctgg  301
       D  G  S  A  P  A  F  H  F  D  P  G  T  G  S  G  I  Y  S  W
302   cttattcaccttcagggtggaggatggtgtgaaaccatttctaactgccaatatcgtgca  361
       L  I  H  L  Q  G  G  G  W  C  E  T  I  S  N  C  Q  Y  R  A
362   cttacggacttatcttcaaaaaatatgcaaaatatatcttattttactggagttttaagt  421
       L  T  D  L  S  S  K  N  M  Q  N  I  S  Y  F  T  G  V  L  S
422   agcgatcctcaacttaatccagacttctacaactggaacagagtcaaggttaagtattgt  481
       S  D  P  Q  L  N  P  D  F  Y  N  W  N  R  V  K  V  K  Y  C
482   gatgggtcatcgtatactggcgatattgaagaagttgatcccgatactaacctccacttt  541
       D  G  S  S  Y  T  G  D  I  E  E  V  D  P  D  T  N  L  H  F
542   agaggagcaagaatttttgaagctatcatggagtatttttaggtcaagggatgatttat  601
       R  G  A  R  I  F  E  A  I  M  E  Y  F  L  G  Q  G  M  I  Y
602   gctcaaaatgccatcctcagtggaacttcagcaggagggttgggtgcaatcatacattgt  661
       A  Q  N  A  I  L  S  G  T  S  A  G  G  L  G  A  I  I  H  C
662   gataagttcagactgtttctcccattaactgctagagtgaaatgcatctcggatgctggt  721
       D  K  F  R  L  F  L  P  L  T  A  R  V  K  C  I  S  D  A  G
722   ttctttgtcgacttagaaacaatatctggcgaaccacttattgaggaaatgtataaaaga  781
       F  F  V  D  L  E  T  I  S  G  E  P  L  I  E  E  M  Y  K  R
782   gttgttgctttgcacggatctgccaagaacctaccactctcttgcactgccttgagctca  841
       V  V  A  L  H  G  S  A  K  N  L  P  L  S  C  T  A  L  S  S
842   gacccaaatttgtgcttcttccctcagtatgtagcaagacatatttgcacaccacttttc  901
       D  P  N  L  C  F  F  P  Q  Y  V  A  R  H  I  C  T  P  L  F
902   atcgtcaactcggcctatgattcttggcagataaacaacagtttggttcctcgagttgct  961
       I  V  N  S  A  Y  D  S  W  Q  I  N  N  S  L  V  P  R  V  A
962   gatcctgacgatgagtggacgtattgcaagaaagatataaatgtatgctcaccaagtcag 1021
       D  P  D  D  E  W  T  Y  C  K  K  D  I  N  V  C  S  P  S  Q
1022  atccaaactctccaagatttcaggttgacatttttggaggcattgaacgaactaggacca 1081
       I  Q  T  L  Q  D  F  R  L  T  F  L  E  A  L  N  E  L  G  P
1082  agtacatcaagaggatatttcatttcctcttgtcattcccaccacggcattgaaatacaa 1141
       S  T  S  R  G  Y  F  I  S  S  C  H  S  H  H  G  I  E  I  Q
1142  aattattggtcctccaaaaactctccaactttagataacaagacgatcgctgaagctgtt 1201
       N  Y  W  S  S  K  N  S  P  T  L  D  N  K  T  I  A  E  A  V
1202  ggagattggtttttcgaccgaagtgggttccaagaagtttattgcccttatccctgtggc 1261
       G  D  W  F  F  D  R  S  G  F  Q  E  V  Y  C  P  Y  P  C  G
1262  aaatttgccagtagagtttctcatttgtactactatttaagattattttttttatta 1321
       K  F  C  Q  *
1322  ttatgtttttttacaagtggagtatctagcaatgcaagaagttagttagctctagtatct 1381

1382  tagatatttgttatccaactactcatgtaattgcttaacattgttcgtcaaacttgcatt 1441

1442  tcgttttcacgttttaaaaaaaaaaaaaaaaaaaaaaaaaaaa 1485
```

Figure S1. cDNA sequence of *NtPAE1* and corresponding predicted amino acid sequence. The numbers represent nucleotide positions in the sequence (1 through 1485). The CDS spans nucleotides 98-1276, producing a predicted protein of 392 amino acids. The start codon ATG and the stop codon TAG are highlighted with underlined nucleotides. The region spanning nucleotides 98-897 (bold and italic highlight) corresponds to the region cloned to produce RNAi transgenic plants. In the amino acid sequence, underlined residues 1-28 correspond to the predicted signal peptide of a secretory pathway substrate cleaved by SPase I (Sec/SPI) (SignalP6 prediction with probability of 0.9998). The red arrow indicates the predicted cleavage site of SPAseI between positions G28 and D29 (SignalP6 prediction with probability of 0.9821). In grey highlight (residues 29-376) is the pectin acetylesterase (PAE) domain (PFAM accession pfam03283), CD-search result with e-value of 0.0. A predicted phosphorylation of Serine 356 is highlited in dark grey (MusiteDeep score 0.634). The predicted active site triad is highlighted in black (amino acids S178, D275 and H342).

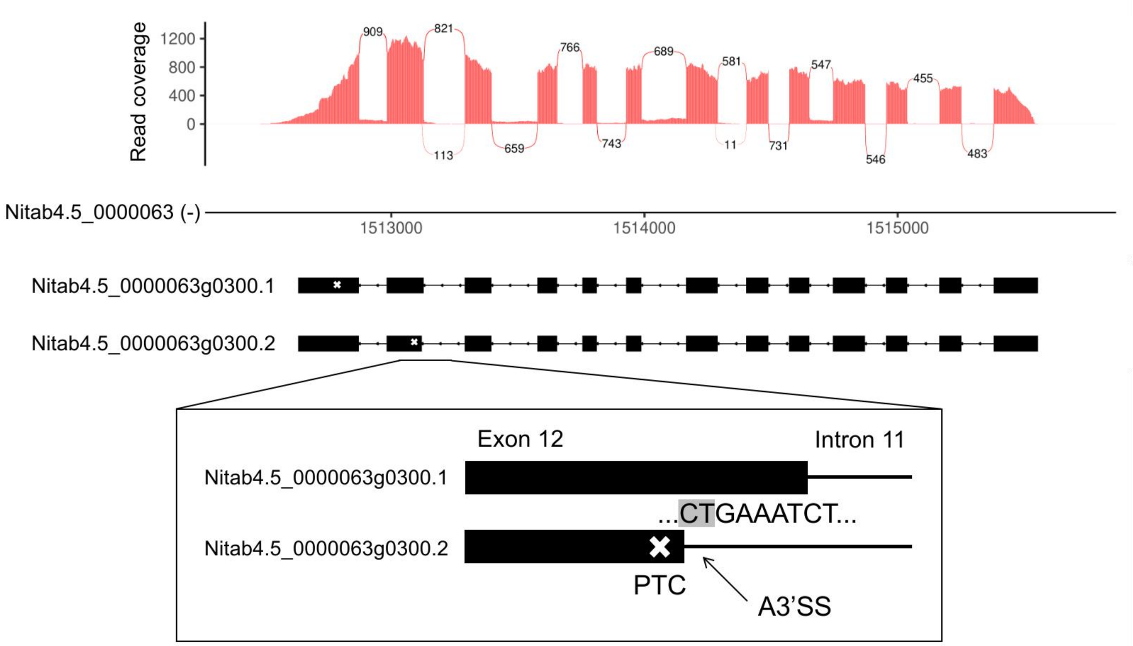

Figure S2. Transcripts of NtPAE1 in the *N. tabacum* genome. RNA-seq coverage of region Nitab4.5_0000063:1512267-1515863 (antisense strand), showing read coverage and exon/intron structure of transcripts from gene Nitab4.5_0000063g0300 (NtPAE1). Transcript Nitab4.5_0000063g0300.1 is the original annotated transcript from Sol Genomics Network database, while Nitab4.5_0000063g0300.2 was manually annotated by predictions made from pubicly available RNA-seq data from flowers of *N. tabacum*. Transcript Nitab4.5_0000063g0300.2 is a result of an alternative 3' splice site (A3'SS, black arrow and grey highlight in the zoomed box) in exon 12, and this event is supported by 113 read junctions. Additionally, the same event is present in *NtPAE1* homologue in *N. tomentosiformis* (gene LOC104098660), which supports our finding. This alternative splicing event creates a premature stop codon (PTC - white "X" in the zoomed box) in exon 12, which could either create a truncated PAE domain in the protein product, or direct the transcribed RNA to the Nonsense Mediated Decay pathway. The graph shows the number of reads mapped to each region of the transcript, while connecting curves represent exon-exon junctions and the number of reads that support them. In the scheme of transcripts, boxes represent exons and lines represent introns.

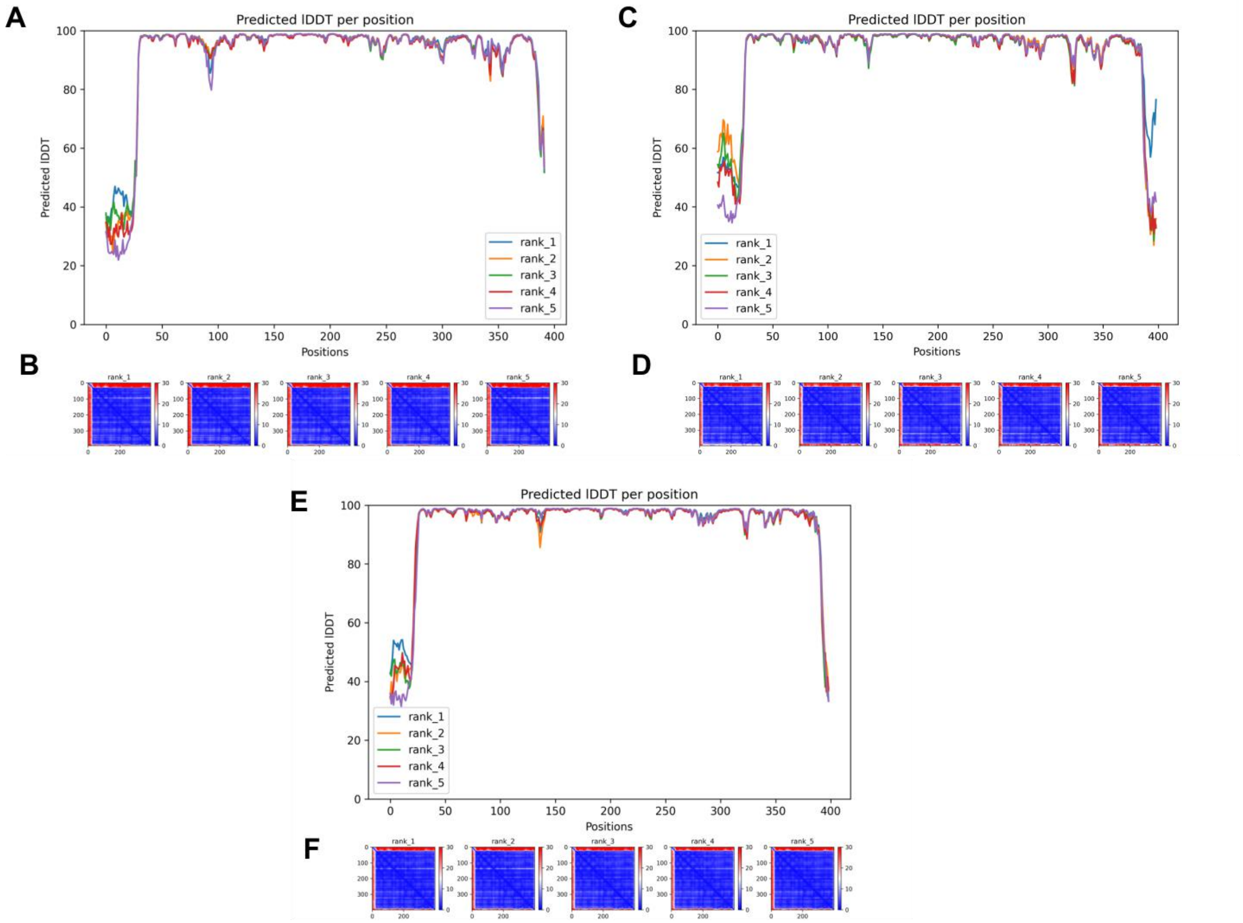

Figure S3. Structure prediction metrics for NtPAE1 (A,B), PvPAE8 (C,D), and VrPAE1 (E,F). A, C, and E: Predicted Local Distance Difference Test (pLDDT) values per amino acid position for five predicted models (rank_1-5). **B, D, and F:** Predicted Aligned Error (PAE) for the five predicted models. y axis = aligned residue, x axis = scored residue. Gradient scale = PAE in Ångstroms.

```
VrPAE1        1  ----METSRKGQWLSVLICVV-LLLKAEGVPVGITFVENAVAKGAVCLDGSPPAYHFHKGSGAGINNWIVHFEGGGWCNNVTSCLSRRD--TRLGSSKKM   93
PvPAE8        1  ----MESVRIIQWLNLVACVL-LLLKAEGSLVPLTLVENALSKGAVCLDGSPPAYHFDKGDGEGINNWIVHMEGGGWCKDADDCLGRKD--TRLGSSKQM   93
NtPAE1        1  MEKVMDCNSILMAAVLFSLLCLWTTTEGDLIVNITYLETAISKGAVCLDGSAPAFHFDPGTGSGIYSWLIHLQGGGWCETISNCQYRAL--TDL-SSKNM   97
HsNOTUM       1  ----------------------------NEDLRLHLL-LNT--SVTCNDGSPAGYYLKESRG--SRRWLLFLEGGWYCFNRENCDSRYDTMRRLMSSRDW   67
Consensus_aa:    ............................+..+.++++..++..+++C.DGS+++++++.++G....+W+++++GG.+C.+.++C..R....++L.SS+++
Consensus_ss:

VrPAE1       94  DTQLSFSGFFSNSKKFNPDFYDWNRIKVRYCDGSSFTGDVEAVDPATNLHFRGARVFAAVVDDLLAKGMKNAQNAIISGCSAGGLAAILNCDRFKSLL--  191
PvPAE8       94  VEDLYFSGILSNEREYNPDFYNWDRVKIRYCDGSSFTGDVEEVDPTNNLYFRGARIFSAVMEDLLAKGMEKAENAILSGCSAGGLTTILHCDNFKALL--  191
NtPAE1       98  QNISYFTGVLSSDPQLNPDFYNWNRVKVKYCDGSSYTGDIEEVDPDTNLHFRGARIFEAIMEYFLGQGMIYAQNAILSGTSAGGLGAIIHCDKFRLFL--  195
HsNOTUM      68  PRTRTGTGILSSQPEENPYWWNANMVFIPYCSSDVWSGASSKSEK-NEYAFMGALIIQEVVRELLGRGLSGAKVLLLAGSSAGGTGVLLNVDRVAEQLEK  166
Consensus_aa:    .+....oG++S++.++NP.+++++.+++.YC++++++G+.+.++..++++F.GA.++..+++.+L++G+..A++++++G+SAGG+++++++D++..+L..
Consensus_ss:

VrPAE1      192  --PRTTKVKCLADAGYFINVKDVSGA-------QRIEEFYSQVVQTHGSAKNLPASCTSRL---RPGLCFFPQNVVSQISTPIFFVNAAYDSWQIKNILA  279
PvPAE8      192  --PSKATVKCVPDAGYFINVEDISGA-------RFIEDYYSKVVSTHGSAKNLPTSCTSKL---SSGLCFFPQYVASHISTPIFIVNSAYDSWQIRNILA  279
NtPAE1      196  --PLTARVKCISDAGFFVDLETISGE-------PLIEEMYKRVVALHGSAKNLPLSCTALSS--DPNLCFFPQYVARHICTPLFIVNSAYDSWQINNSLV  284
HsNOTUM     167  LGYPAIQVRGLADSGWFLDNKQYRHTDCVDTITCAPTEAIRRGIRYWNG--VVPERCRRQFQEGEEWNCFFGYKVYPTLRSPVFVVQWLFDEAQLT-VDN  263
Consensus_aa:    ....+++V++++D+G+F++.++++............++++++++.++++..++P.+C+......+...CFF++.V+.++.+P+F+V+.++D++Q++...+
Consensus_ss:

VrPAE1      280  PGAADPHGTWRECKLDIKKCSSNQLSAMQVFRTDFLRAFGAVGNSPSKGHFIDGCYAHCQTGTQETWLRNDSPVLGSTTIAKAVGDWYYD-----R--KP  372
PvPAE8      280  PGAADPSDNWHSCKLDLAKCSPDQLSIVQGFKKEFEKAVSVVGDSSSNGMFINSCYDHCQTESQETWSKSDSPHLANTTISKAVGNWFYD-----R--SP  372
NtPAE1      285  PRVADPDDEWTYCKKDINVCSPSQIQTLQDFRLTFLEALNELGPSTSRGYFISSCHSHHGIEIQNYWSSKNSPTLDNKTIAEAVGDWFFD-----R--SG  377
HsNOTUM     264  V--------------HLPVQEGLRLYIQNLGRELRHTLK-----DVPASFAPACLSHEIIIRSH-WT---DVQVKGTSLPRALHCWDRSLHKTPLKGCP  339
Consensus_aa:    +..............+..++.++..++Q++..++.+++......++.+.F+++C++H..+..++.W....++.+.++++++A+.+W..+.........+
Consensus_ss:

VrPAE1      373  FKQID-CAY-PCNPTCHNRIFDQNERPDV  399
PvPAE8      373  FHHVD-CNY-PCNPTCQNTVSDLKDHPGI  399
NtPAE1      378  FQEVY-CPY-PCGKFCQ------------  392
HsNOTUM     340  VHLVDSCPWPHCNPSCPTG----------  358
Consensus_aa:    ++++..C++..C+..C.............
Consensus_ss:
```

Figure S4. Structural alignment of predicted plant PAE structures and HsNOTUM. Bold amino acid residues are conserved among the four sequences. Plus signs indicate similar amino acid chemical properties. Dots represent non-conserved sites. Dark grey areas represent consensus alpha-helices, and light gray areas represent beta-strands. Active site residues are indicated with black arrows and black highlighted residues..

	NOTUM	NtPAE1	VrPAE1	PvPAE8
NOTUM	1			
NtPAE1	0.86001	1		
VrPAE1	0.86643	0.92203	1	
PvPAE8	0.86834	0.92456	0.97593	1

Figure S5. TM-align scores of pairwise structural alignments between plant PAEs and HsNOTUM. Scores with 0.5 < TM-score < 1.0 means structures assume the same fold (Zhang and Skolnick, 2005).
