# Supplementary material for: Silencing of a Pectin Acetylesterase (PAE) Gene Highly Expressed in Tobacco Pistils Negatively Affects Pollen Tube Growth
Source: Plants (Basel). 2023 Jan 10;12(2):329. doi: 10.3390/plants12020329 (PMC9864977; doi:10.3390/plants12020329)
Supplement: Supplementary file 1 [file plants-12-00329-s001.zip › Table S1 - primer descriptions.pdf]

Table S1. Oligonucleotides used for PCR amplifications, gene cloning and expression analyses. Small letters represent attB sites. Grey boxes mark the start and stop codons of NtPAE1. Letters in bold and underlined are the Kozak consensus sequence.

Primer name	Primer sequence (5'-3')
PAE-attB1	agcaggcttc**accATGG**AGAAAGTGATGGATTG
PAE-attB2	gaaagctgggtcCTACTGGCAAAATTTGCCAC
Ri-PAE-attB2	gaaagctgggtcAGTGGTGTGCAAATATGTCTTGC
BP1	GGGGACAAGTTTGTACAAAAAAGCAGGCTTC
BP2	GGGGACCACTTTGTACAAGAAAGCTGGGTC
RT-qPCR PAE-Fw	AGGAGGGTTGGGTGCAATCATACA
RT-qPCR PAE-Rv	GCAGATCCGTGCAAAGCAACAACT
GAPDH - Fw	GCATCTTTGATGCCAAGGCTGGAA
GAPDH - Rv	TCGAGTGCTGTAGCCCATTTCGTT
Actin - Fw	AGATGATGCTCCAAGGGCTGTGTT
Actin - Rv	CCATGCCAACCATCACACCAGT
